# Supplementary material for: FUS unveiled in mitochondrial DNA repair and targeted ligase-1 expression rescues repair-defects in FUS-linked motor neuron disease
Source: Nat Commun. 2024 Mar 9;15:2156. doi: 10.1038/s41467-024-45978-6 (PMC10925063; doi:10.1038/s41467-024-45978-6)
Supplement: Supplementary file 1 — Supplementary Information [file 41467_2024_45978_MOESM1_ESM.pdf]

Supplementary Information

FUS Unveiled in Mitochondrial DNA Repair and Targeted Ligase-1 Expression Rescues Repair-Defects in FUS-Linked Motor Neuron Disease

Manohar Kodavati[1#], Haibo Wang[1#], Wenting Guo[2,3,4], Joy Mitra[1], Pavana M. Hegde[1], Vincent Provasek[1,5], Vikas H Maloji Rao[1], Indira Vedula[6], Aijun Zhang[6,7], Sankar Mitra[1], Alan E. Tomkinson[8], Dale J. Hamilton[6,7], Ludo Van Den Bosch[2,9], Muralidhar L. Hegde[1,10*]

[1]Division of DNA Repair Research within the Center for Neuroregeneration, Department of Neurosurgery, Houston Methodist Research Institute, Houston, TX 77030, USA.
[2]KU Leuven-Department of Neurosciences, Experimental Neurology and Leuven Brain Institute (LBI), Leuven, 3000, Belgium.
[3]Stem Cell Institute, Department of Development and Regeneration, KU Leuven, Leuven, 3000, Belgium.
[4]INSERM, UMR-S1118, Mécanismes Centraux et Périphériques de la Neurodégénérescence, Université de Strasbourg, CRBS, 67000 Strasbourg, France.
[5]College of Medicine, Texas A&M University, College Station, TX 77843, USA.
[6]Center for Bioenergetics, Houston Methodist Research Institute, Houston, TX 77030, USA.
[7]Department of Medicine, Houston Methodist, Weill Cornell Medicine affiliate, Houston, TX, 77030, USA.
[8]Departments of Internal Medicine, and Molecular Genetics and Microbiology and University of New Mexico Comprehensive Cancer Center, University of New Mexico, Albuquerque, NM, 87131, USA.
[9]VIB, Center for Brain & Disease Research, Laboratory of Neurobiology, Leuven, Belgium.
[10]Department of Neuroscience, Weill Cornell Medical College, New York, NY 10065, USA.

#These authors contributed equally.
*Correspondence to: mlhegde@houstonmethodist.org

Supplementary Material accompanying this paper includes three Tables and six Figures.

Supplementary Figure 1

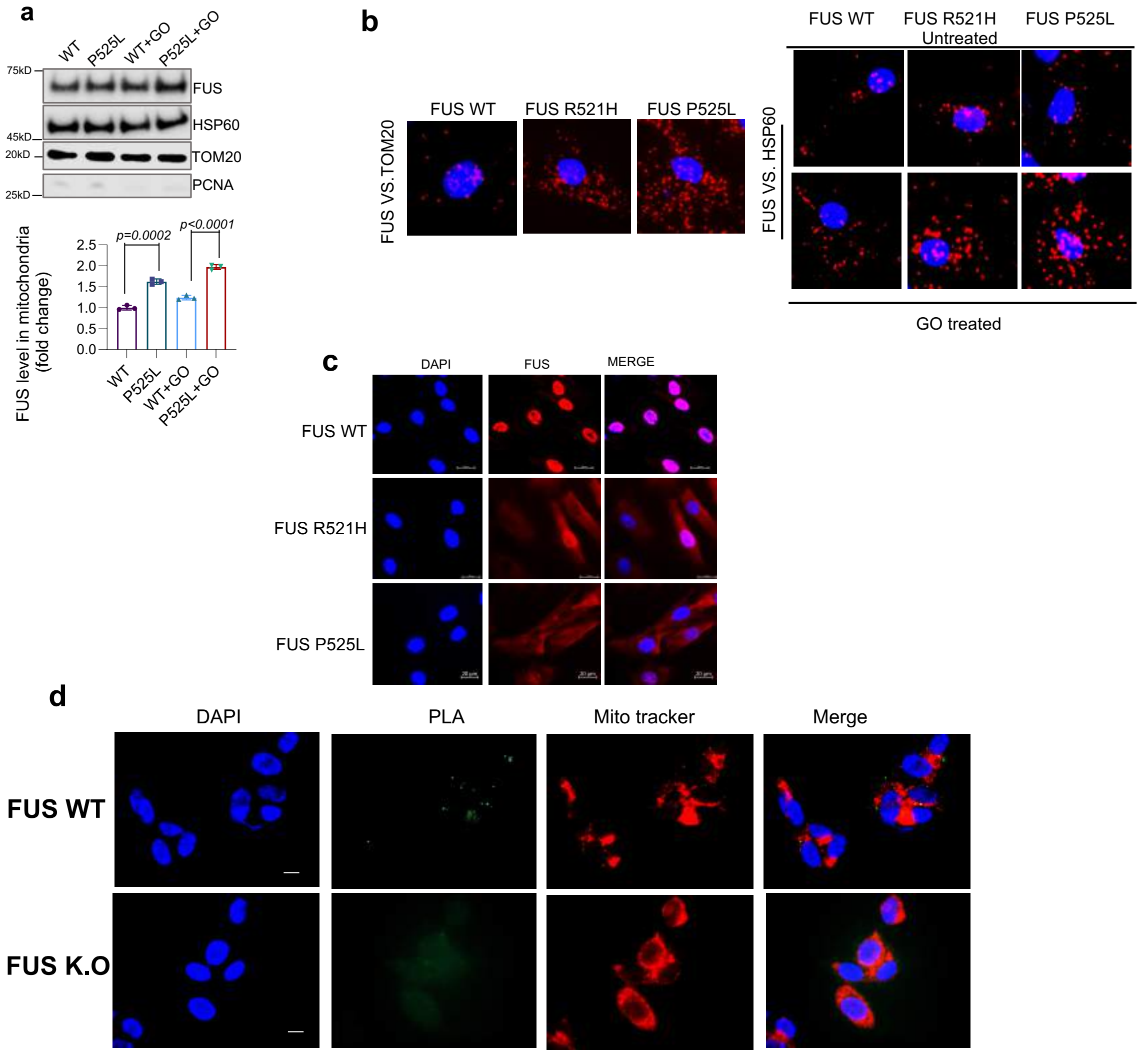

Supplementary Figure 1: Effect of FUS mutation on its cellular localization. (Related to Fig. 1)

a. FUS localization to mitochondria in control and patient fibroblasts and effect of GO on FUS recruitment.
b. Magnified images of FUS VS TOM20 and HSP60 (Red), Nucleus stained with DAPI
c. IF of endogenous FUS localization in patient derived fibroblasts, Scale bar=20µm.
d. PLA (green) between FUS and Tom20 in FUS KO SHSY-5Y cells mitochondria stained with Mito tracker red and nuclei stained with DAPI, Scale bar=20µm

All statistical analysis were performed using two-sided student-t test in graph pad prism software.

Source data are provided as a Source Data file.

Supplementary Figure 2

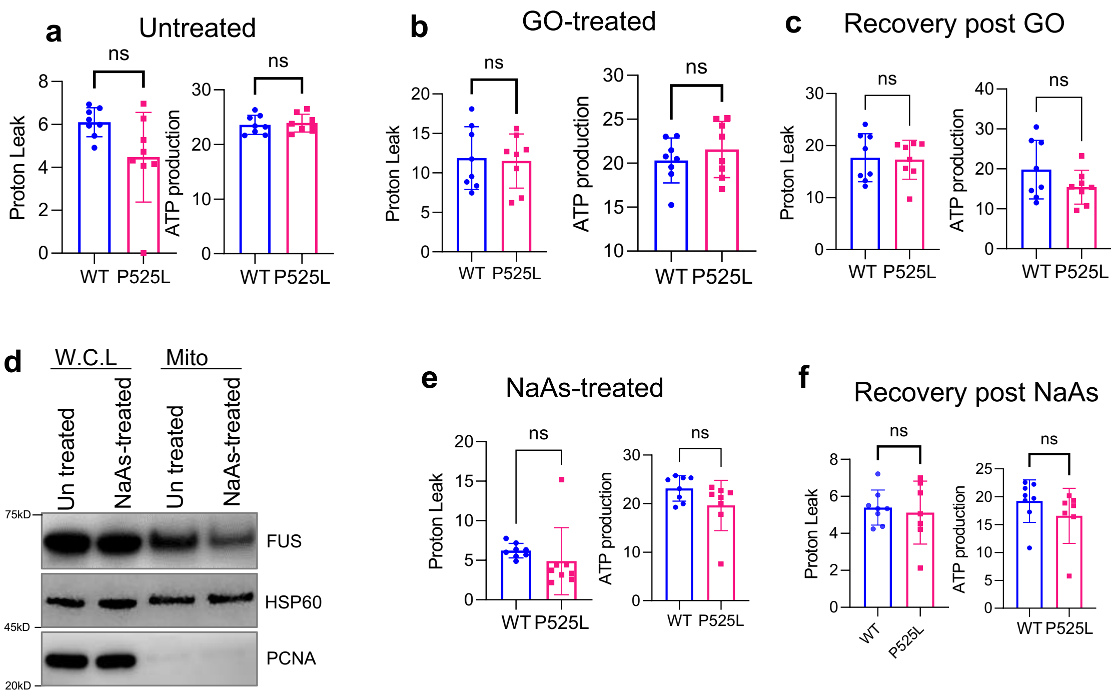

Supplementary Figure 2: Assessment of mitochondrial functional parameters using seahorse assay. (Related to Fig. 2)

a-c. Quantification of proton leak and ATP production capacity between WT and P525L fibroblasts using seahorse assay, a corresponds to untreated, b and c corresponds to GO treatment and recovery after GO treatment respectively.

d. IB showing localization of FUS to mitochondria after sodium arsenite treatment along with loading controls HSP60 and PCNA.

e and f. Corresponds to Proton leak and ATP production capacity quantification between WT and P525L fibroblasts using seahorse assay, e corresponds to arsenite treatment and f represents recovery after the treatment.

All statistical analysis were performed using two-sided student-t test in graph pad prism software.

Source data are provided as a Source Data file.

Supplementary Figure 3

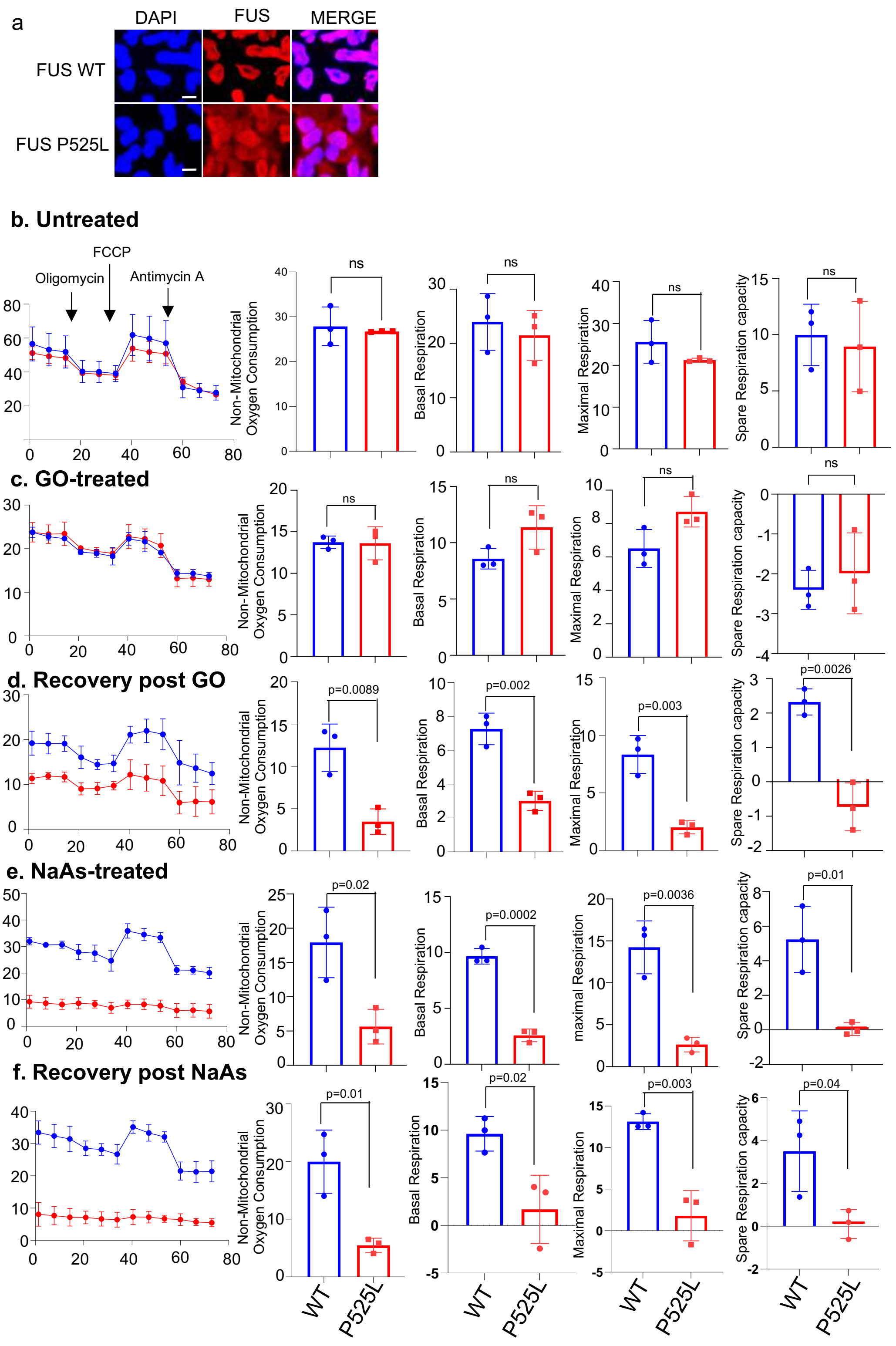

Supplementary Figure 3: Mitochondrial respiration comparison between FUS WT and FUS P525L NPSC by seahorse assay. (Related to Fig. 2)

a. FUS localization in untreated FUS WT and P525L NPSC cells using IF, Scale bar=5µm.

b-f. b untreated, GO treated c, recovery after GO treatment d, sodium arsenite treated e and recovery after sodium arsenite treatment f. Oxygen consumption rate (OCR) determined throughout the mitochondrial respiration test in control and patient derived P525L NPSC. Arrows indicate the time when mitochondrial inhibitors were added to the media to assess respiratory parameters. Non-mitochondrial oxygen consumption was determined by measuring difference between total oxygen consumption and antimycin A and rotenone treatment induced reduction in oxygen consumption, maximal respiration was assessed as difference between oxygen consumption following mitochondria uncoupling by FCCP and rotenone, antimycin A treatment and spare respiratory capacity was determined by subtracting basal respiration from maximal respiration. All statistical analysis were performed using two-sided student-t test in graph pad prism. Source data are provided as a Source Data file.

Supplementary Figure 4

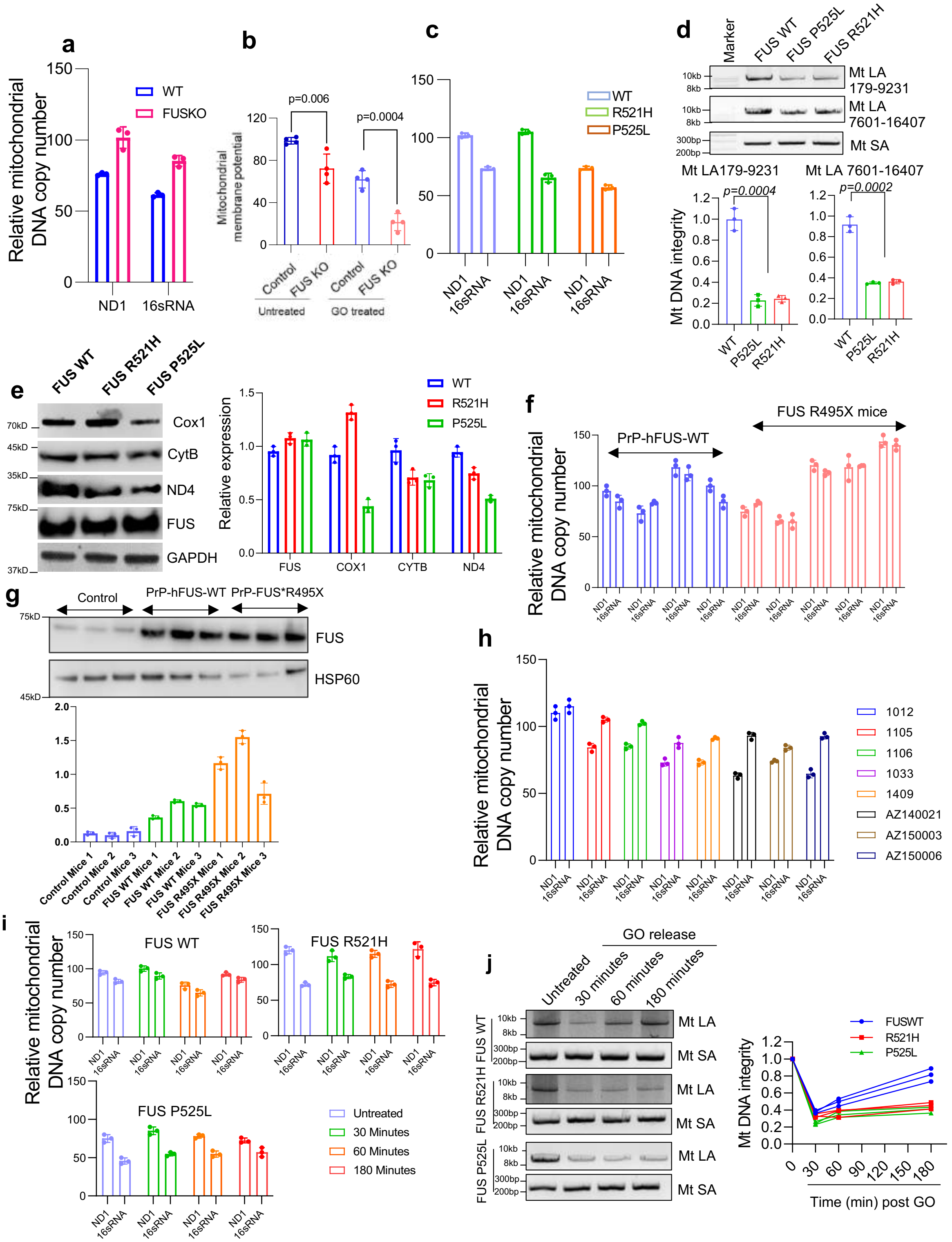

Supplementary Figure 4: Determination of relative mitochondrial DNA content for samples used in LA-PCR assay, FUS localization to mouse mitochondria and effect of FUS pathology on mitochondrially encoded genes. (Related to Fig. 3)

a. Represents relative mitochondrial DNA content in HEK293 cells WT and FUSKO cells,
b. Changes in membrane potential in FUS KO cells in comparison to FUS WT HEK 293 cells with and without GO treatment.
c. Relative mitochondrial DNA content in control and patient fibroblasts
d. LA-PCR to determine mitochondrial DNA integrity of Control and patient derived fibroblasts.
e. Effect of FUS pathology on expression of mitochondrially encoded genes from iPSC derived motor neurons.
f. Relative mitochondrial DNA content comparison between mouse brains.
g. Localization of FUS in mitochondrial extracts of mouse brain.
h. Relative mitochondrial DNA comparison between control and patient tissues.
i. Comparison of relative mitochondrial content in FUS WT and Mutant fibroblasts after GO treatment and recovery.
j. Comparison of DNA repair capacity between FUS WT and FUS mutant fibroblast cells using LA-PCR assay and the resulting product are run on 1% agarose gel.

All statistical analysis were performed using two-sided student-t test in graph pad prism software.

Source data are provided as a Source Data file.

Supplementary Figure 5

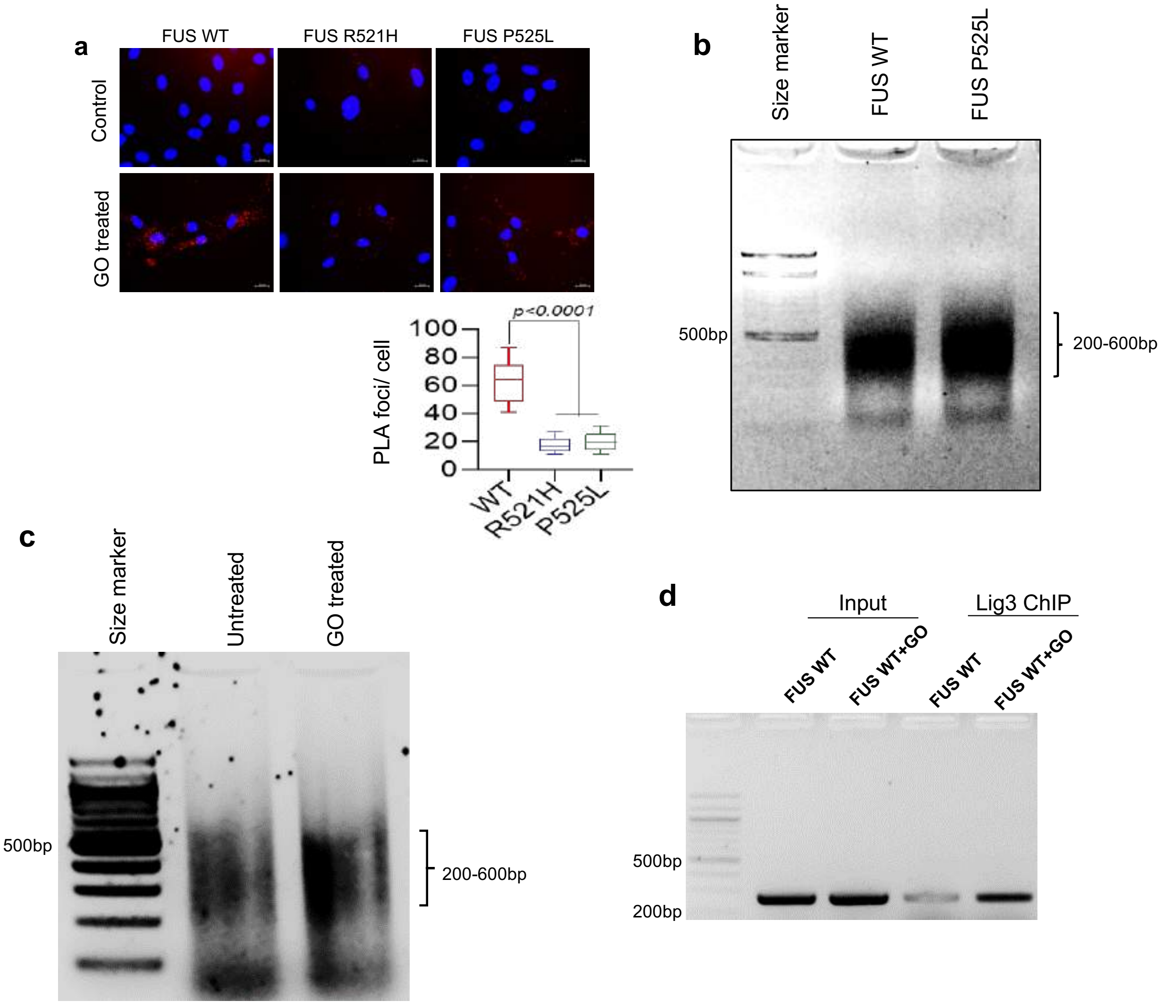

Supplementary Figure 5: FUS pathology causes defective FUS-Lig3 interaction and effects Lig3 recruitment to mtDNA. (Related to Fig. 4)

a. PLA of FUS-Lig3 in patient derived fibroblasts, PLA foci (Red), Nucleus stained with DAPI, foci were counted from 25 cells Scale bar=20µm.

b. Representative image of agarose gel separation of the input DNA fragment size between 250bp and 600bp used in ChIP assay.

c and d. represents mitochondrial ChIP assay performed in FUS WT fibroblast cell with and with out GO treatment.

All statistical analysis were performed using two-sided student-t test in graph pad prism software.

Source data are provided as a Source Data file.

Supplementary Figure. 6

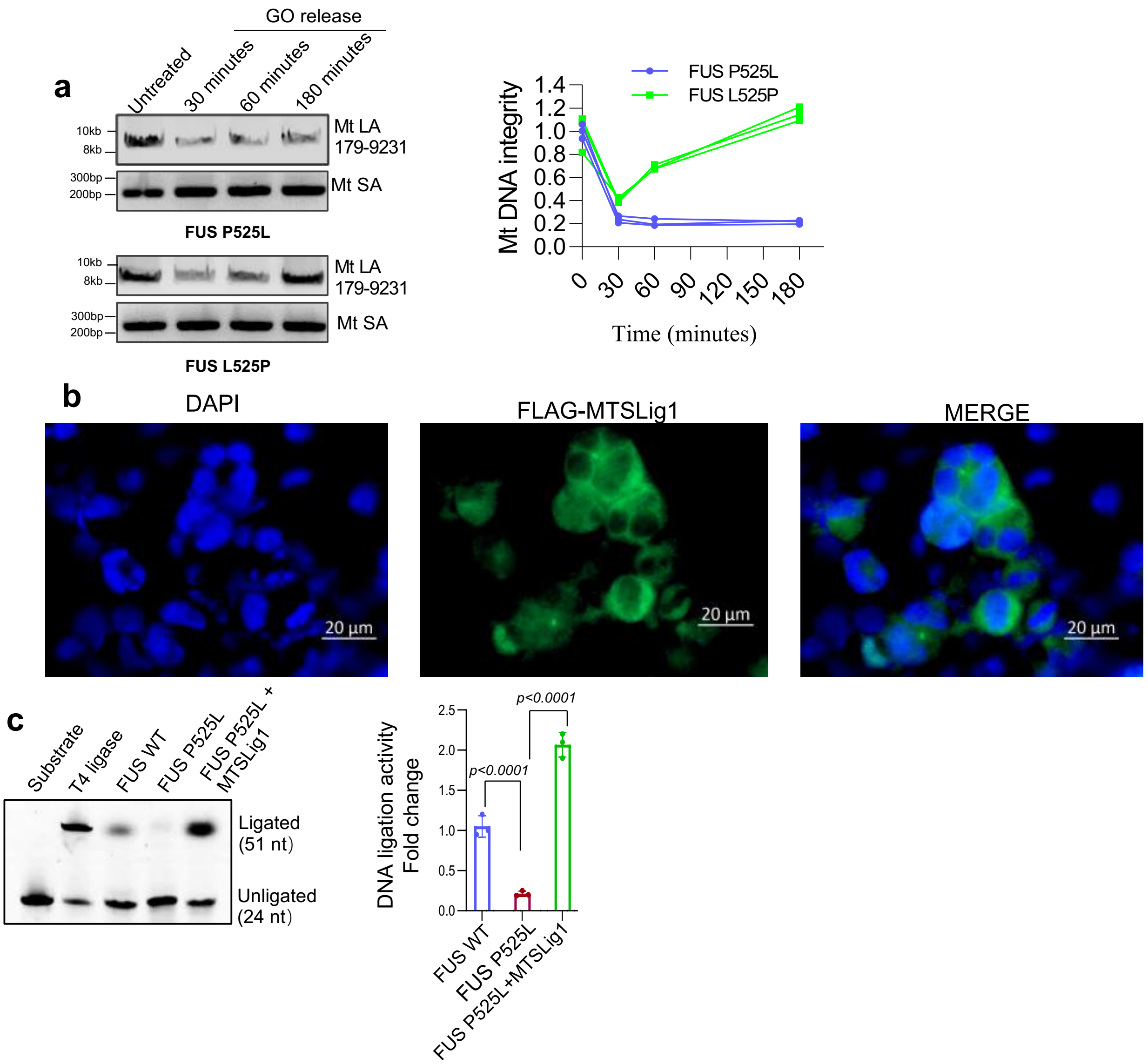

Supplementary Figure 6: FUS P525L causes DNA repair defects and can be rescued by mutation correction. (Related to Fig. 5)

a. LA-PCR-based DNA damage repair kinetic analysis. Genome DNA extracted from iPSC derived motor neurons with FUS P525L and FUS L525P isogenic cells at indicated time points after release from exposure to GO (100 ng/ml) for 1 h. Amplification products analyzed by agarose gel electrophoresis and Pico green assay.

b. IF of FLAG-MTSLig1 localization in HEK293 cells, IF was performed by anti-FLAG antibody. DAPI staining indicates nucleus. Scale bar = 20 µm

c. In Vitro ligase activity assay performed in control and FUS P525L fibroblast cells with and without MTS-Lig1 expression.

All statistical analysis were performed using two-sided student-t test in graph pad prism software. Source data are provided as a Source Data file.

Supplementary Table 1. Clinical features of ALS patients and matched controls.

Control and ALS patients (from VA Biorepository) clinical features						
	Case #		Age	Gender	Ethnicity	PMI-cr (hours)
Control	C-1	100012	81	F	White	<4.0
	C-2	110005	62	M	Hispanic/Latino	2.75
	C-3	110006	68	M	White	1.5
ALS patients	P-1	100033	63	M	White	2.33
	P-2	140009	76	M	White	0.87
	P-3	AZ140021	63	M	White	0.40
	P-4	AZ150003	88	M	White	3.25
	P-5	AZ150006	88	M	White	3.00

Supplementary Table 2. Primers used in this study

1	Human nuclear short amplicon Forward primer	TGC TGT CTC CAT GTT TGA TGT ATC T
2	Human nuclear short amplicon Forward primer	TCT CTG CTC CCC ACC TCT AAG T
3	Mouse nuclear short amplicon Forward primer	GCCAGCCTCTCCTGATTTTAGTGT
4	Mouse nuclear short amplicon Forward primer	GGGAACACAAAAGACCTCTTCTGG
5	Human mitochondrial long amplification (179) Forward primer	TTACAGGCGAACATACTTAC
6	Human mitochondrial long amplification (9231) Reverse primer	GATAGGCATGTGATTGGTG
7	Human mitochondrial long amplification (7601) Forward primer	CAAGTAGGTCTACAAGACG
8	Human mitochondrial long amplification (16407) Reverse primer	GGATATTGATTTCACGGAGG
9	Human ND1 Forward primer	AAGAACACCTCTGATTACTCCTGCC
10	Human ND1 Reverse primer	GTTGTGTAGAGTTCAGGGGAGAGTGCG
11	Human 16sRNA Forward primer	AGGTAGAGGCGACAAACCTACCGAGC
12	Human 16sRNA Reverse primer	TTAATCTGACGCAGGCTTATGCGGAGG
13	FLAG-MTS Ligase1 forward primer	CCGGCGC TCG AGCATGTCCGTCCTGACGCCGCTG CTGCTGCGGGGCTTGACAGGCTCGGCCCGGCGGCT CCCAGTGCCGCGCGCCAAGATCCATTCGTTGATG CAG CGA AGT ATC ATG TC
14	DNA Ligase1 reverse primer	CCGGCTCGAGTTA GTA GGT ATC TTC AGG GTC
15	Mouse mitochondrial long amplification (2372) Forward primer	GCCAGCCTGACCCATAGCCATATTAT
16	Mouse mitochondrial long amplification (13337) Reverse primer	GAGAGATTTTATGGGTGTATTGCGG
17	Mouse ND1 Forward primer	CTAGCAGAAACAAACCGGGC
18	Mouse ND1 Reverse primer	TGCTCGGATCCATAGGAATG
19	Mouse 16sRNA Forward primer	CCGCAAGGGAAAGATGAAAGAC
20	Mouse 16sRNA Reverse primer	TCGTTTGGTTTCGGGGTTTC

Supplementary Table 3. Types of unique mutations

Type of variation	FUS R521H	FUS P525L	FUS K.O	Patient
A/C	15	8	8	
A/G	52	48	28	15
A/T	9	8	5	1
C/A	7	7	6	3
C/G	9	3	3	
C/T	134	134	166	19
G/A	123	135	123	21
G/C	1	2	1	1
G/T	3	4	5	
T/A	3	6	6	1
T/C	75	49	47	18
T/G	9	7	7	1
Delins	28	7	9	
Insertions	27	24	28	12
