# Supplementary material for: FUS unveiled in mitochondrial DNA repair and targeted ligase-1 expression rescues repair-defects in FUS-linked motor neuron disease
Source: Nat Commun. 2024 Mar 9;15:2156. doi: 10.1038/s41467-024-45978-6 (PMC10925063; doi:10.1038/s41467-024-45978-6)
Supplement: Supplementary file 3 — Reporting Summary [file 41467_2024_45978_MOESM3_ESM.pdf]

nature portfolio

Corresponding author(s): Dr. Muralidhar Hegde

Last updated by author(s): 01/23/2024

Reporting Summary

Nature Portfolio wishes to improve the reproducibility of the work that we publish. This form provides structure for consistency and transparency in reporting. For further information on Nature Portfolio policies, see our Editorial Policies and the Editorial Policy Checklist.

Statistics

For all statistical analyses, confirm that the following items are present in the figure legend, table legend, main text, or Methods section.

n/a	Confirmed	
☐	☒	The exact sample size (n) for each experimental group/condition, given as a discrete number and unit of measurement
☐	☒	A statement on whether measurements were taken from distinct samples or whether the same sample was measured repeatedly
☐	☒	The statistical test(s) used AND whether they are one- or two-sided<br>*Only common tests should be described solely by name; describe more complex techniques in the Methods section.*
☒	☐	A description of all covariates tested
☒	☐	A description of any assumptions or corrections, such as tests of normality and adjustment for multiple comparisons
☐	☒	A full description of the statistical parameters including central tendency (e.g. means) or other basic estimates (e.g. regression coefficient) AND variation (e.g. standard deviation) or associated estimates of uncertainty (e.g. confidence intervals)
☐	☒	For null hypothesis testing, the test statistic (e.g. F, t, r) with confidence intervals, effect sizes, degrees of freedom and P value noted<br>*Give P values as exact values whenever suitable.*
☒	☐	For Bayesian analysis, information on the choice of priors and Markov chain Monte Carlo settings
☒	☐	For hierarchical and complex designs, identification of the appropriate level for tests and full reporting of outcomes
☒	☐	Estimates of effect sizes (e.g. Cohen's d, Pearson's r), indicating how they were calculated

Our web collection on statistics for biologists contains articles on many of the points above.

Software and code

Policy information about availability of computer code

Data collection	LI-COR Odyssey, FLUO VIEW FV3000 imaging system
Data analysis	PolyPhen-2, NCBI Genome Work bench, graph pad, prism 8

For manuscripts utilizing custom algorithms or software that are central to the research but not yet described in published literature, software must be made available to editors and reviewers. We strongly encourage code deposition in a community repository (e.g. GitHub). See the Nature Portfolio guidelines for submitting code & software for further information.

Data

Policy information about availability of data

All manuscripts must include a data availability statement. This statement should provide the following information, where applicable:

- Accession codes, unique identifiers, or web links for publicly available datasets
- A description of any restrictions on data availability
- For clinical datasets or third party data, please ensure that the statement adheres to our policy

All the data generated in this study is included in the published article and supplementary files. The sequencing data have been submitted to the Genome variation map database, data can be accessed in the following link (https://ngdc.cncb.ac.cn/gvm/getProjectDetail?Project=GVM000579)
All reagents generated in this study are available from the lead contact with a material transfer Agreement. Original data for the study can be found in source data file. Further information and requests for resources and reagents should be directed to Lead contact Dr. Muralidhar Hegde (mlhegde@houstonmethodist.org)

Research involving human participants, their data, or biological material

Policy information about studies with human participants or human data. See also policy information about sex, gender (identity/presentation), and sexual orientation and race, ethnicity and racism.

Reporting on sex and gender	Autopsied spinal cord tissue samples from ALS patients were procured from the Department of Veterans' Affairs (VA) Biorepository in the USA. The study utilized a total of 8 spinal cord samples, 7 of which were from male subjects.
Reporting on race, ethnicity, or other socially relevant groupings	The five patient samples were all sourced from white male patients. Among the control samples, two are male: one is Hispanic/Latino and the other is white. The third control sample is from a white female.
Population characteristics	See above
Recruitment	Autopsied spinal cord tissue samples from ALS patients were procured from the Department of Veterans' Affairs (VA) Biorepository in the USA.
Ethics oversight	These studies were carried out in compliance with the ethical standards of the Department of Veterans' Affairs and the institutional review boards at the Houston Methodist Research Institute in Houston, Texas.

Note that full information on the approval of the study protocol must also be provided in the manuscript.

Field-specific reporting

Please select the one below that is the best fit for your research. If you are not sure, read the appropriate sections before making your selection.

☒ Life sciences ☐ Behavioural & social sciences ☐ Ecological, evolutionary & environmental sciences

For a reference copy of the document with all sections, see nature.com/documents/nr-reporting-summary-flat.pdf

Life sciences study design

All studies must disclose on these points even when the disclosure is negative.

Sample size	The sample size was determined in accordance with the standards prevalent in the fields of biochemistry and cell biology, aiming for a minimum of N=3. The specific sample sizes are indicated in the main text figures, the methods sections, or the figure legends.(PMID: 30206235,PMID: 31067307,PMID: 30770445)
Data exclusions	No data were excluded from the analysis in this study.
Replication	Immunofluorescence and western blots were performed 3 times. All replication attempts were successful and yielded similar observations in each instance. One representative result has been presented. All the experiments were performed a minimum of three times and the exact number is presented in the figure legends for each figure
Randomization	In the study, samples were allocated randomly.
Blinding	Blinding was not utilized in this study. The data was obtained from instrument-based and software-based analyses.

Reporting for specific materials, systems and methods

We require information from authors about some types of materials, experimental systems and methods used in many studies. Here, indicate whether each material, system or method listed is relevant to your study. If you are not sure if a list item applies to your research, read the appropriate section before selecting a response.

Materials & experimental systems

n/a	Involved in the study	
☐	☒	Antibodies
☐	☒	Eukaryotic cell lines
☒	☐	Palaeontology and archaeology
☐	☒	Animals and other organisms
☒	☐	Clinical data
☒	☐	Dual use research of concern
☒	☐	Plants

Methods

n/a	Involved in the study	
☒	☐	ChIP-seq
☒	☐	Flow cytometry
☒	☐	MRI-based neuroimaging

Antibodies

Antibodies used

Rabbit anti-FUS (Cat# A300–302A) and anti-Lig1 (A301-136A) antibodies were procured from Bethyl Laboratories, Inc. Mouse anti-FLAG antibody (A8592) was obtained from Sigma-Aldrich, and mouse anti-Lig3 antibody (Cat# ab587) was purchased from Abcam. Mouse anti-Tom20 (SC-17764), anti-HSP60 (SC-13115) and anti-PCNA (SC-56) antibodies were procured from Santa Cruz Biotechnology. Rabbit COX-1 (13393-1-AP), CYTB (55090-1-AP) and ND4 (26763-1-AP) were obtained from Protein tech. Fluorescent secondary antibodies, Alexa Fluor 488 anti-mouse (Cat# A28175), and Texas Red anti-rabbit antibody (Cat# T-2767) were obtained from Life Technologies. The antibodies were diluted at 1:1000 for western blotting, 1:500 for immunofluorescence and 1:100 for PLA.

Validation

All primary antibodies were verified, and experiments utilizing these antibodies were repeated at least three times. All the antibodies were purchased from commercial sources, and their validations can be found on their respective websites. Additionally, it's worth mentioning that most of these antibodies are routinely used in our lab and have been validated through a gene knockdown strategy in cell extracts.
Rabbit anti-FUS (Cat# A300–302A for PLA, IF and WB) Whole cell lysate (50microgram) from HeLa, HEK293T, and mouse NIH3T3 cells prepared using NETN lysis buffer. Antibody: Affinity purified rabbit anti-FUS antibody A300-302A used at concentration of 1:1000 for WB and IP (Immunoprecipitation). This antibody was previously used in our other publication for IF and PLA (Haibo Wang., et al, 2018 Nature communications 9, Article number:368(2018).
Rabbit anti-Lig1 (A301-136A) is shown to detect human Ligase 1 by western blot at 1:1000 dilution.
Mouse anti-FLAG antibody (A8592) was suggested to use as 1:1000 dilution by the manufacturer.
Mouse anti-LigaseIII (ab587) was shown to have species reactivity to human samples and this antibody was previously used in our study (Haibo Wang., et al, 2018 Nature communications 9, Article number: 368(2018).
Tom20 Antibody (F-10): sc-17764. Western blot analysis of Tom20 expression in Jurkat, Caki-1, A549, HeLa and Raji whole cell lysates.
HSP 60 Antibody (B-9): sc-271215. Western blot analysis of HSP 60 expression in HeLa, and KNRK cell lysates.
PCNA Antibody (PC10) HRP: sc-56 HRP. Direct western blot analysis of PCNA expression in HCT-116, MOLT-4 and C6 whole cell lysates.

Eukaryotic cell lines

Policy information about cell lines and Sex and Gender in Research

Cell line source(s)

Primary human fibroblasts were obtained from skin biopsies of ALS patients and controls with the approval of the ethical committee of the University Hospitals Leuven (S50354).FUS R521H sample obtained from 71- year old female patient, FUS P525L mutation sample obtained from 17-year old male patient, and Control sample was obtained from unaffected female parent at age 54. Samples were collected in the year 2012.
HEK293 cells were obtained from ATCC cell line repository.

Authentication

No additional authentication was performed in this study. These cell lines have been previously reported in several publications including our Nature Communication Publication in 2018 (PMID 30206235)

Mycoplasma contamination

All cell lines are routinely analyzed by PCR for ruling out mycoplasma contamination and found negative

Commonly misidentified lines
(See ICLAC register)

No commonly misidentified cell lines was used

Animals and other research organisms

Policy information about studies involving animals; ARRIVE guidelines recommended for reporting animal research, and Sex and Gender in Research

Laboratory animals

FUS WT (Strain#:017916) and R495X (Strain#:017928) transgenic mice were obtained from the Jackson Laboratory repository. Animals were propagated and genotyped following the guidelines provided by the Jackson Laboratory. The Mice used in our study were around 12 month old , mouse brain tissue used in the study obtained from male animals.

Wild animals

No Wild animals were used in this study

Reporting on sex

The Mice used in our study were around 12 month old , mouse brain tissue used in the study obtained from male animals.

Field-collected samples

No field collected samples were used in the study

Ethics oversight

The animal care and use in the research program at the Houston Methodist Research Institute (HMRI) adhere to all relevant federal and state laws on animal care. The program also complies with the guidelines for the use of laboratory animals as per the Institutional Animal Care and Use Committee (IACUC). The approved IACUC protocol number for this study is IS00006797.
The mice were kept under constant conditions (21 ± 1°C; 60% humidity) with a 12-hour light/dark cycle, and were given unrestricted access to food and water. The mice were weaned at 21 days and genotyped via ear biopsy.

Note that full information on the approval of the study protocol must also be provided in the manuscript.
